# Expression Profiles of the Ovine *IL18* Gene and Association of Its Polymorphism With Hematologic Parameters in Hu Lambs

**DOI:** 10.3389/fvets.2022.925928

**Published:** 2022-06-30

**Authors:** Deyin Zhang, Xiaoxue Zhang, Fadi Li, Yuan Zhao, Xiaolong Li, Jianghui Wang, Liming Zhao, Xiaobin Yang, Yukun Zhang, Dan Xu, Jiangbo Cheng, Wenxin Li, Changchun Lin, Bubo Zhou, Weimin Wang

**Affiliations:** ^1^The State Key Laboratory of Grassland Agro-Ecosystems, Key Laboratory of Grassland Livestock Industry Innovation, Ministry of Agriculture and Rural Affairs, Engineering Research Center of Grassland Industry, Ministry of Education, College of Pastoral Agriculture Science and Technology, Lanzhou University, Lanzhou, China; ^2^College of Animal Science and Technology, Gansu Agricultural University, Lanzhou, China

**Keywords:** *IL18* gene, expression profiles, hematological traits, heritability, SNPs, association, sheep

## Abstract

Hematological traits are important indexes to evaluate health status and immunological conditions in human and livestock. In this study, we measured the hematologic indexes of 819 male Hu lambs and performed the descriptive statistical analysis. The results showed the coefficients of variation of partial indexes were >10%, and the heritability for mean erythrocyte volume (MCV), white blood cell count (WBC), hemoglobin concentration (HGB), hematocrit (HCT), and red blood cell (RBC) distribution-standard deviation (RDW_SD) were medium to high, ranging from 0.17 to 0.43. In addition, Interleukin 18 (*IL18*), as an important regulator of both innate and acquired immune responses, was selected as candidate gene and subjected to the expression profile analysis, single nucleotide polymorphism (SNP) scanning and association analysis by using quantitative real-time PCR (qRT-PCR), PCR amplification, Sanger sequencing, and KASP genotyping. The results of qRT-PCR indicated that *IL18* is predominantly expressed in lymph and lung compared with that in the other tested tissues. In addition, three novel polymorphisms (g. 24991544 A > G, g. 24991651 A > G, and g. 24991749 C > T) were identified in *IL18*, and the three SNPs were in a strong linkage state. Therefore, only a SNP was genotyped and performed association analysis in the enlarged experimental population, the result of association analysis demonstrated that the polymorphism g. 24991651 A > G was significantly associated with RBC, MCV, MCHC, and RDW_CV. These results will provide the reference values and the novel genetic markers of hematological parameters in sheep.

## Introduction

Sheep (*Ovis aries*) are important economic animals, which provide people with valuable agricultural commodities (1). In the past, the breeding program of sheep mainly focused on the genetic improvement of economic traits such as litter size (2–4), growth traits (5, 6), feed efficiency (7). However, with the emergence of antibiotic prohibition policy and people are highly concerned about healthier livestock products (8). The health-related traits and welfare of animal will play an important role in breeding programs, because the health status is closely related to the animal performance and welfare as well as the profitability for farms (9). While hematologic parameters are general indicators for the evaluation of the immunological status and health conditions of various organs, and can serve as biomarkers to reflect pathological condition and disease severity in human and livestock. Therefore, the identification of genetic determinants underlying hematological parameter indexes variability will contribute to elucidate the biological mechanisms of different diseases related to these indexes.

In the immune system, hematologic traits include the following three components: platelets, leukocytes (white blood cells, WBCs), and erythrocytes (red blood cells, RBCs), which are important indexes for evaluating the immune capacity of animal (10). To date, the studies on candidate gene and quantitative trait loci (QTL) for hematologic parameters have been reported in human (11) and in a few livestock species such as pigs (12, 13), poultry (14), and cattle (15). In pigs, Zhang et al. identified 44 significant SNPs associated with 11 hematological traits in Chinese Sutai pigs using genome-wide association analysis (12). Luo et al. reported 77, 10, and 24 significant SNPs were identified for the platelet, leukocyte and erythrocyte traits in swine using genome-wide associations (16). In chicken, a genome wide association analysis of immune traits identified nine loci on chromosome 4 related to hematologic parameters (14). However, few studies have reported the candidate genes and QTLs association with hematologic traits in sheep. Interleukin 18 (*IL18*, also known as IFN-γ-inducing factor) is a member of IL-1 family of cytokines, and first discovered for its interferon-γ-inducing properties, which play crucial roles in the regulation of innate and acquired immune response (17, 18), and *IL18* is a pleiotropic cytokine playing a role in in immune, infectious, and inflammatory diseases (19), while hematologic indexes are important indicators of immune function in animal and have been commonly used as biomarkers of disease and disease severity. The report on polymorphisms in *IL18* gene have mainly focused on human (20), there are not on studies associating the genetic polymorphism of *IL18* gene with hematologic indexes of sheep.

Hence, in this study, we measured the hematologic indexes of 819 male Hu lambs and estimated their genetic parameters. Simultaneously, *IL18* gene was considered as candidate gene, the expression features of *IL18* gene were analyzed, and the polymorphisms for *IL18* gene were identified to perform an association analysis of hematologic indexes and different genotypes. These results will provide the reference information and the potential genetic markers of hematological parameters in sheep.

## Methods

### Ethics Statement

All animal studies were performed in strict accordance with the guidelines proposed by the Regulation of the Standing Committee of Gansu People's Congress. The collection of blood and tissue samples as well as the experimental procedures involved in this study was approved by the Ethics Committee of the College of Pastoral Agriculture Science and Technology of Lanzhou University.

### Samples and Phenotypic Data Collection

**Figure 1:**
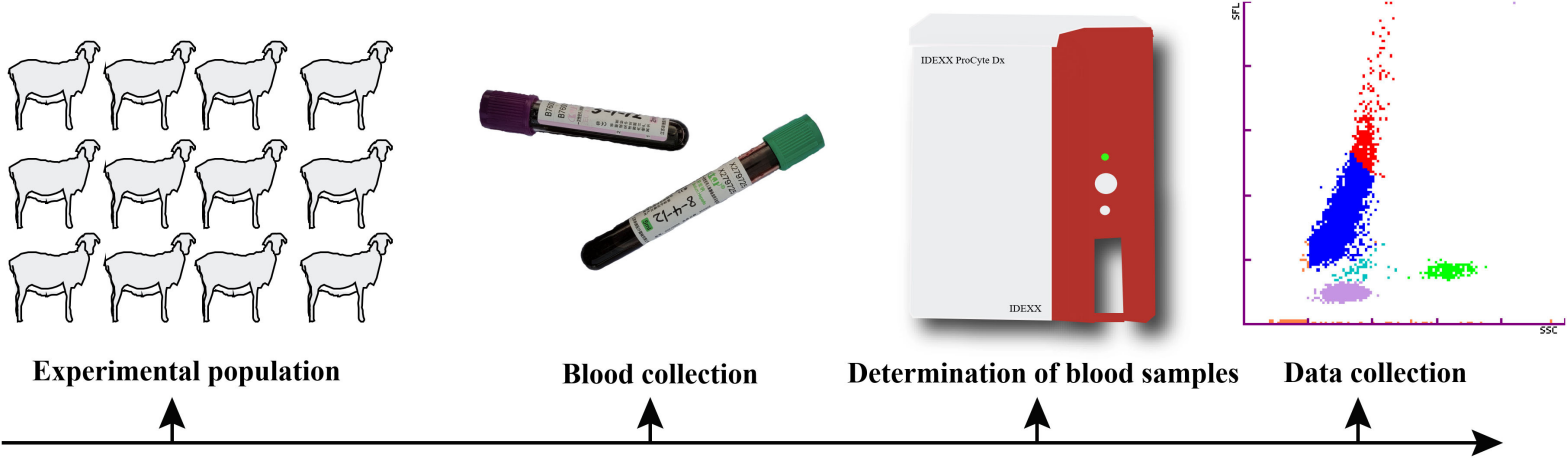
Schematic diagram of the experimental protocol.

The male Hu lambs with healthy and detailed pedigrees (*n* = 819) used in this study were obtained from four Hu sheep farms as follows: Zhongsheng Huamei Sheep Industry Development Co. Ltd.; Shandong Runlin Sheep Industry Co. Ltd.; Changxing Yongsheng Husbandry Co. Ltd.; and Hangzhou Pangda Agricultural Development Co. Ltd. All lambs were transferred to Minqin Defu Agriculture Co. Ltd., 56 days after weaning, and reared indoor in a single pen with free access to clean water and pellet feed, of which the pellet feed is commodity material from Gansu Sanyangjinyuan Husbandry Co. Ltd. Throughout the period, all lambs used the same immunization procedures, feeding regime and management methods according to our previous report (21). At 180 days of age, two whole blood samples were collected from the jugular vein of each individual in the morning according to the experimental protocol was shown in Figure 1, of which one tube (5 ml) of whole blood containing sodium heparin is stored at −20°C until DNA extraction and the other a tube (2 ml) of whole blood containing EDTA-K_2_ is used for measuring the hematological parameter indexes by using Hematology Analyzer (ProCyte Dx, IDEXX, Westbrook, ME, USA). The instrument was installed and operated in the veterinary clinic at the Minqin Defu Agriculture Co. Ltd; the samples collected were measured within 10 min of collection according to sheep standard. After slaughtered, a small portion tissue sample from the same section of the heart, liver, spleen, lung, kidney, rumen, duodenum, lymph, tail fat, and longissimus dorsi were collected from each sheep, briefly washed with PBS and placed in freeze tube (2 ml), and immediately preserved in liquid nitrogen. At the end of the slaughter experimental procedure, the tissue samples placed in liquid nitrogen were transferred to the laboratory, then stored at −80°C until further use.

### DNA Extraction, RNA Extraction, cDNA Synthesis

Genomic DNA was extracted from the above blood samples using an EasyPure Blood Genomic DNA Kit (TransGen Biotech, Beijing, China) following the manufacturer's instructions, and the extracted DNA were used as a template for subsequent PCR amplification and KASP genotyping studies. Four male Hu sheep were randomly selected at 6 months of age from the experimental population (*n* = 819) for collecting the tissue sample; total RNA was extracted from 10 tissues (heart, liver, spleen, lung, kidney, rumen, duodenum, lymph, tail fat, and longissimus dorsi) using the TRIzol reagent (TransGen, Beijing, China) in accordance with the manufacturer's instruction. First-strand cDNA was synthesized from 2-μg total RNA using an Evo M-MLV RT Kit with gDNA Clean for qPCR (Accurate Biotechnology Co., Ltd, Hunan, China) following the manufacturer's protocols.

### Tissue Expression Analysis of *IL18* Gene

The expression levels of *IL18* were detected in different tissues by using quantitative real-time PCR (qRT-PCR). Specific primers (*IL18*-expression-F and *IL18*-expression-R) for *IL18* gene (Accession No. NM_001009784.3) as listed in Table 1. The qRT-PCR was performed on Roche Light Cycler 480 system (Roche Applied Science) and SYBR Green PCR Master Mixture (TaKaRa), β-actin was used as the internal control gene, the reaction systems and procedures were as described in our previous study (7). The 2^−ΔΔCT^ method was used to calculate the expression level (22).

**Table 1:** Details of primer sequences used for qRT-PCR and PCR amplification.

**Primer name**	**Primer sequence (5^**′**^-3^**′**^)**	**GenBank accession number**	**Annealing temperature (**°**C)**	**Size (bp)**
*IL18*-expression-F	CATACGAAATTTGAACGACCA	XM_012095263.3	60	154
*IL18*-expression-R	TACAGCCAGACCTCTAGTGA			
*β-actin*-F	TCCGTGACATCAAGGAGAAGC	NM_001009784.3	60	256
*β-actin*-R	CCGTGTTGGCGTAGAGGT			
*IL18*-SNP-F	CTGGCCTTTCTAACACTCC	NC_040266.1	55	605
*IL18*-SNP-R	AAGAATGAGAACTGTTGCCTA			

### Polymerase Chain Reaction Amplification, Mutations Detection, and Genotyping

Polymerase chain reaction primer (*IL18*-SNP-F and *IL18*-SNP-R) was designed for *IL18* gene (Accession No. NC_040266.1) using Oligo 7.0 (Table 1), the PCR fragments were amplified by using the above primer pairs and 20 mixed DNA samples selected randomly from the 819 individual genomic DNA, the reaction volume containing 12.4 μl of 2× Tsingke Master Mix, 0.8 μl of each forward and reverse primer, 10 μl of H_2_O, and 1 μl mixed DNA. The reaction conditions were as follows: A temperature at 94°C for 5 min, followed by 35 cycles (94°C, 55°C, and 72°C for 30 s), with a final extension 72°C for 5 min. The 1.5% agarose gel electrophoresis was used to check the amplified products, then, the PCR product was sequenced using Sanger sequencing by Tsingke (Xi'an, China). The sequencing results were blast to by DNAstar software to identify the candidate SNPs. Finally, the competitive allele-specific fluorescence resonance energy transfer (FRET)-based PCR were used to genotype the SNPs identified within *IL18* gene according to a previously studied method (23), the KASP primer pairs were designed and listed in Table 2. Briefly, KASP genotyping is based on competitive allele-specific PCR amplification of target sequences and endpoint fluorescence genotyping (KASPar™) assays. The universal KASP Master Mix and SNP-specific KASP Assay mix were added to DNA samples. The thermal cycling was then performed, followed by an end-point fluorescent read. Finally, to complete the allele identification, we performed competitive annealing of the two allele-specific forward primers, each primer having a unique tail sequence that corresponding to a distinct labeled FRET cassette in the Master Mix, one labeled with FAM™ dye and the other with HEX™ dye, allowing quantification and scoring of the different SNPs.

**Table 2:** Primers used for KASP detection.

**Primer's purpose**	**Primer name**	**Primer sequence (5'−3')**
KASPar	*IL18*_SNP1_AlleleX	GAAGGTGACCAAGTTCATGCTCACTTTCCCCATTCGTAGTGTGATT
	*IL18*_ SNP1_AlleleY	GAAGGTCGGAGTCAACGGATTCTTTCCCCATTCGTAGTGTGATC
	*IL18*_ SNP1_Common	GTATTTTTCAAAAGTCCCTGTTTTCTATTTTTGG
	*IL18*_ SNP2_AlleleX	GAAGGTGACCAAGTTCATGCTAGGAGATCATAGGCTTTATATGGGTA
	*IL18*_ SNP2_AlleleY	GAAGGTCGGAGTCAACGGATTGGAGATCATAGGCTTTATATGGGTG
	*IL18*_ SNP2_Common	CAACCCTTGTCCCTCTGGCATTC
	*IL18*_ SNP3_AlleleX	GAAGGTGACCAAGTTCATGCTTTCTGGAAGTAGTGGAGAAGGGA
	*IL18*_ SNP3_AlleleY	GAAGGTCGGAGTCAACGGATTCTGGAAGTAGTGGAGAAGGGG
	*IL18*_ SNP3_Common	AACCTCACTGGTCTGTTTGCTCTAC

### Statistical Analysis

The multiple traits model was constructed based on average information (AI) restricted maximum likelihood method (REML) using Hiblup software (https://www.hiblup.com/tutorials) to estimate variance components and heritability. The pedigree was used for constructing the matrix of relationships between the individuals. The linkage disequilibrium analysis and construction of haplotypes were performed using Haploview 4.2, the allele and genotypic frequency and Hardy-Weinberg equilibrium (HWE) testing *p*-value were calculated using SNPassoc package of R language (version 4.1.0). The heterozygosity (He), homozygosity (Ho), polymorphic information content (PIC), and effective allele number (Ne) were directly calculated by using online software (http://www.msrcall.com/Gdicall.aspx) based on the allele frequency, genotypic frequency and number. The association of the different genotype with hematological parameter indexes, including RBC (red blood cell count), HGB (hemoglobin concentration), HCT (hematocrit), MCV (mean erythrocyte volume), MCH (mean erythrocyte hemoglobin content), MCHC (mean erythrocyte hemoglobin concentration), RDW_SD (red blood cell distribution-standard deviation), RDW_CV (red blood cell distribution–coefficient of variation), and WBC (white blood cell count) was performed by using SPSS 17.0 software. The following general liner model was adopted: *Y*_*ijkl*_ = μ + Genotype_*i*_ + Batch_*j*_ + Father_*k*_ + Mother_*l*_ + ε_*ijkl*_, where *Y*_*ijkl*_ represents the traits observation value, μ is the population mean, Genotype_*i*_ represents the effect of genotype, Batch_*j*_ is the effect of the *j*th batch (*j* = 1, 2, …, 4), Father_*k*_ and Mother_*l*_ are the family effects within breed, ε_*ijkl*_ is the random error. One-way ANOVA was performed to estimate the difference of different genotypes, the result was expressed as “mean ± standard error (SE).” A *p* < 0.05 was considered as the statistically significant criterion. The bar graph and violin plot were drawn using the ggplot packages of R software (version 4.1.0).

## Results

### Descriptive Statistics of Hematologic Traits

**Figure 2:**
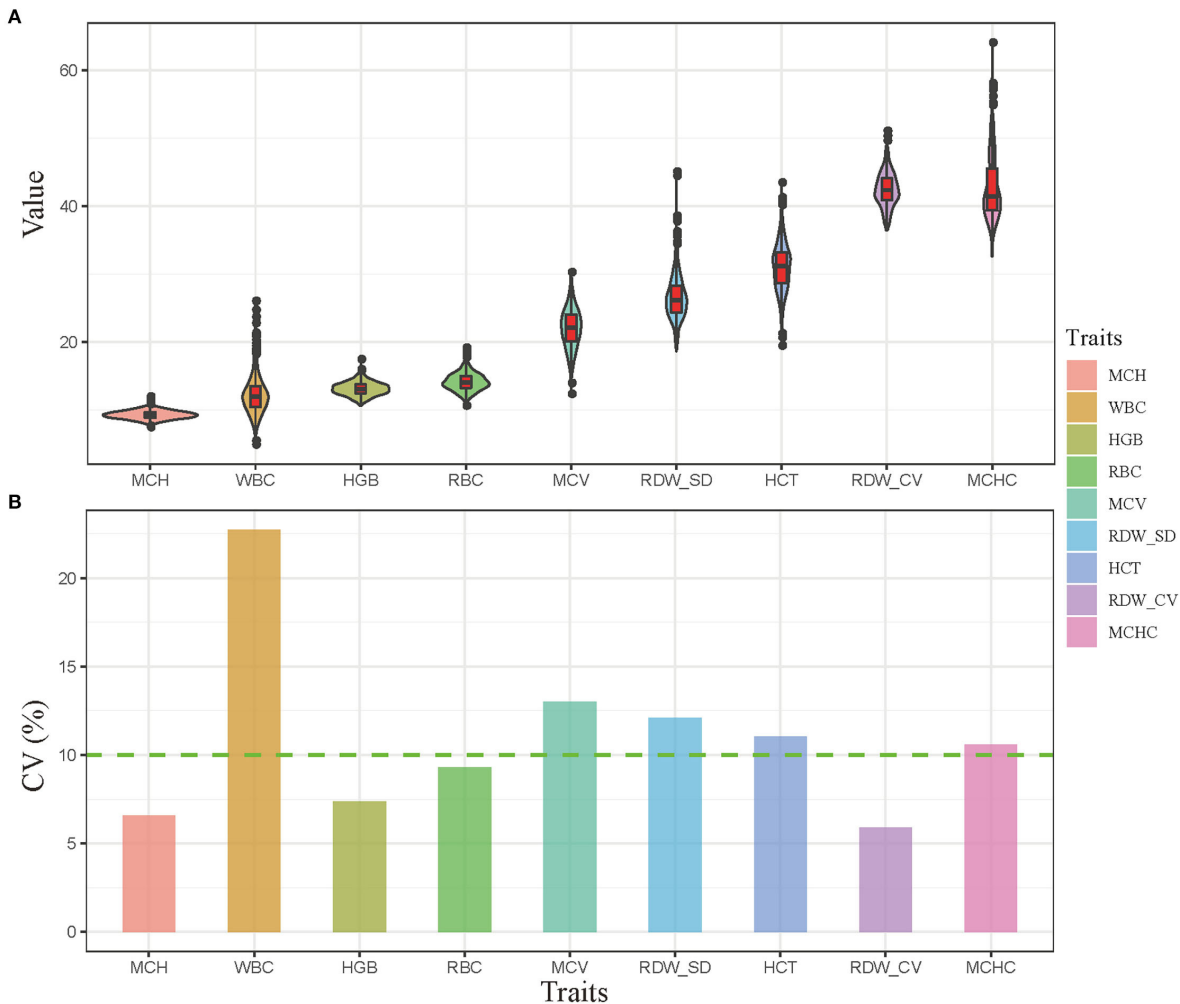
Descriptive statistics for hematologic parameters. **(A)** The violin plot of hematologic indexes. **(B)** The coefficient of variation of hematologic indexes.

In this study, the hematologic traits for 819 male Hu lambs were measured at 180 days of age according to the schematic diagram of the experimental protocol (Figure 1). The descriptive statistical for the phenotype of hematological parameter indexes are shown in Figure 2, the average values of the RBC (red blood cell count), HGB (hemoglobin concentration), HCT (hematocrit), MCH (mean erythrocyte hemoglobin content), WBC (white blood cell count), MCV (mean erythrocyte volume), RDW_CV (red blood cell distribution-coefficient of variation), and RDW_SD (red blood cell distribution-standard deviation) were within the reference values, and the coefficient of variation of WBC, HCT, MCV, MCHC, and RDW-SD were >10%. In addition, the estimates of the variance components and heritability for hematologic traits using pedigree relationship matrices, the results are shown in Table 3, the heritability for five hematological parameter indexes (MCV, WBC, HGB, HCT, and RDW_SD) were medium to high, ranging from 0.17 to 0.43 (Table 3).

**Table 3:** Variance components and estimates of heritability for hematologic traits.

**Traits**	** $\sigma_{a^{2}}$ **	** $\sigma_{e^{2}}$ **	***h* ^2^**
RBC (M/ul)	0.23 ± 0.59	2.49 ± 0.54	0.08 ± 0.22
HGB (g/dl)	0.37 ± 0.35	1.18 ± 0.32	0.24 ± 0.22
HCT (%)	2.74 ± 1.80	7.99 ± 1.65	0.26 ± 0.16
MCV (fl)	0.81 ± 0.60	4.07 ± 0.57	0.17 ± 0.12
MCH (pg)	0.07 ± 0.05	0.39 ± 0.05	0.15 ± 0.11
MCHC (g/dl)	1.77 ± 1.11	5.66 ± 0.99	0.24 ± 0.14
RDW_SD (fl)	4.37 ± 1.43	5.84 ± 1.27	0.43 ± 0.13
RDW_CV (%)	0.49 ± 0.69	5.80 ± 0.69	0.08 ± 0.11
WBC (K/ul)	1.73 ± 1.53	7.72 ± 1.43	0.18 ± 0.16

*σ${}_{{\text{a}}^{2}}$, additive effect variance component; σ${}_{{\text{e}}^{2}}$, residual variance component; h^2^, heritability; RBC, red blood cell count; HGB, hemoglobin concentration; HCT, hematocrit; MCV, mean erythrocyte volume; MCH, mean erythrocyte hemoglobin content; MCHC, mean erythrocyte hemoglobin concentration; RDW_SD, red blood cell distribution-standard deviation; RDW_CV, red blood cell distribution-coefficient of variation; WBC, white blood cell count*.

### Expression of *IL18* in Sheep With Different Tissues

**Figure 3:**
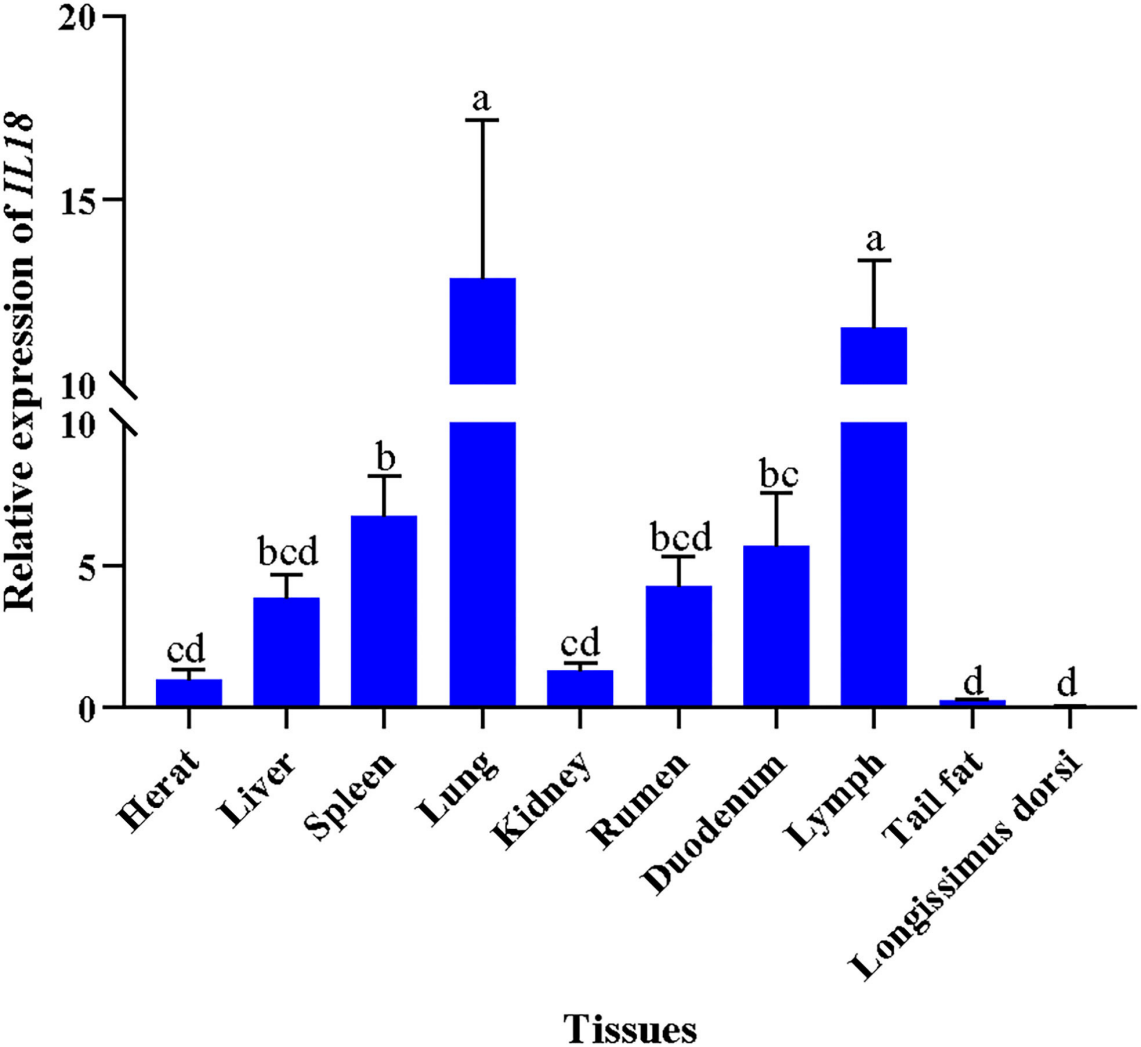
The expression levels of *IL18* gene in 10 tissues of the Hu sheep. Different letters indicate significant difference (*p* < 0.05).

The expression of *IL18* gene in heart, liver, spleen, lung, kidney, longissimus dorsi, rumen, duodenum, lymph, and tail fat tissues was detected in Hu sheep at 6 months age. Taking the expression of the *IL18* gene in the heart as a reference, the result is shown in Figure 3, the *IL18* gene was widely expressed in ten tissues, and *IL18* is predominantly expressed in lymph and lung compared with the others tested tissues.

### Identification of Polymorphism Within Sheep *IL18* Gene

**Figure 4:**
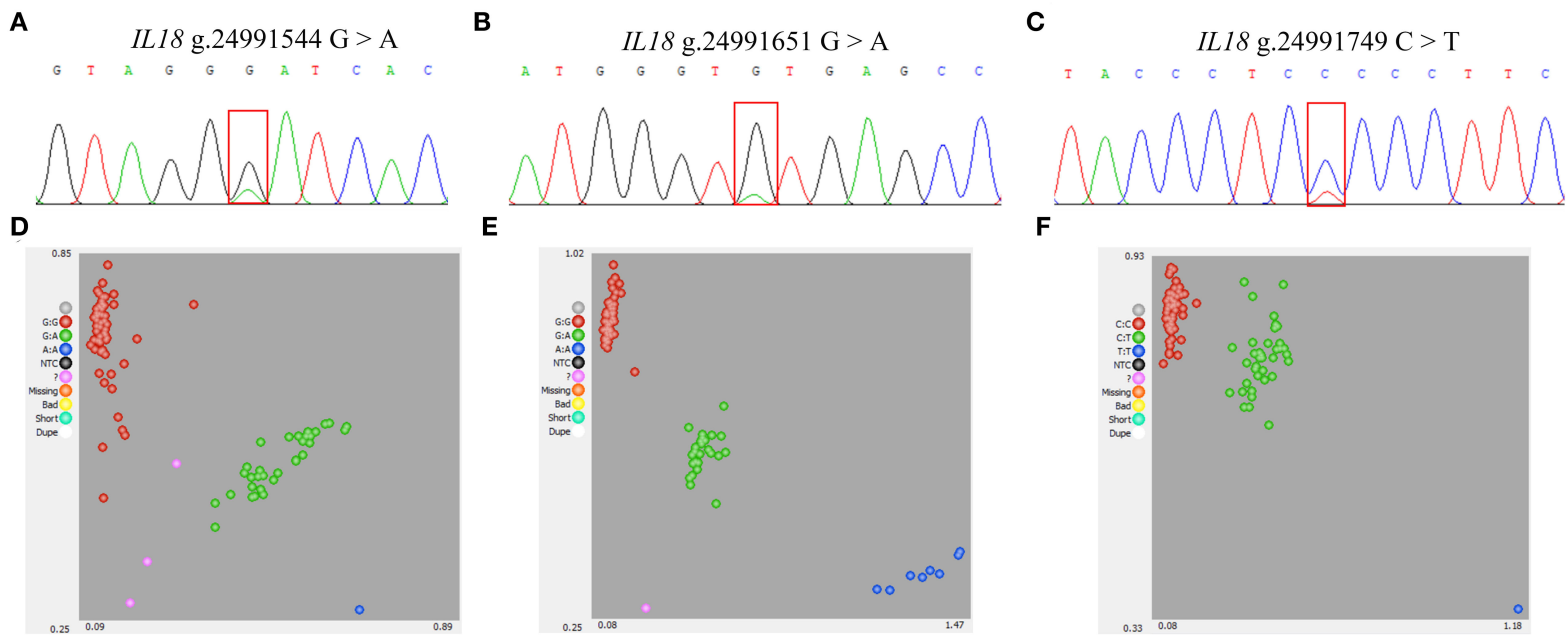
Identification and genotyping of SNPs within ovine *IL18* gene. **(A–C)** Sequence chromatograms of three SNPs in the sheep *IL18* gene. **(D–F)** KASPar genotyping assay results of three positions of *IL18* gene.

The sequence of 605 bp fragment of the *IL18* gene was amplified using mixed genomics DNA from 20 Hu sheep as templates by PCR, a total of three SNP in the amplified sequence were identified by sequencing the PCR products. The sequenced peak images for three SNPs are shown in Figures 4A–C.

### Genotyping, Linkage Disequilibrium, and Population Genetics Analysis

**Figure 5:**
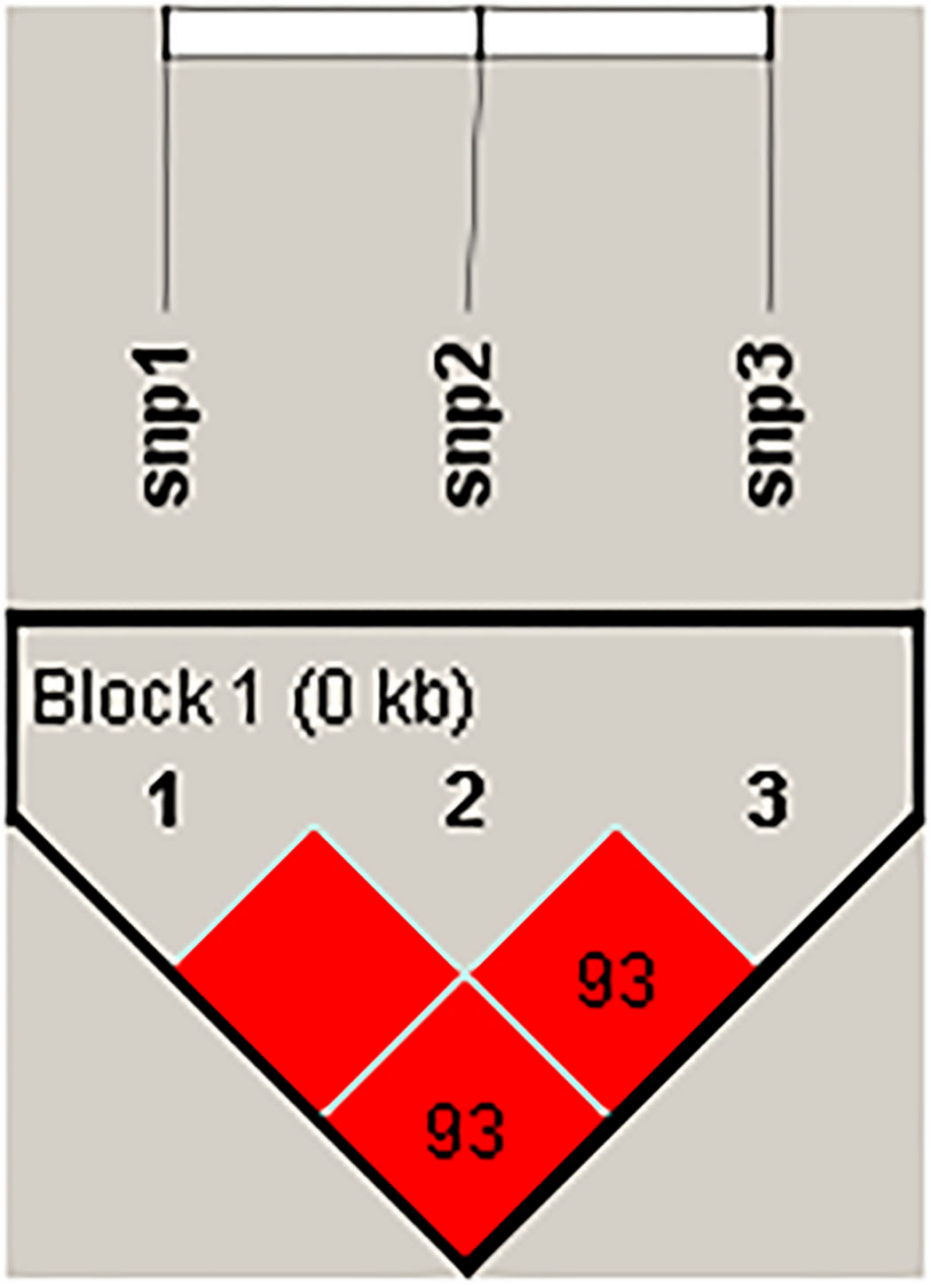
Linkage disequilibrium analysis of three SNPs of *IL18*.

**Figure 6:**
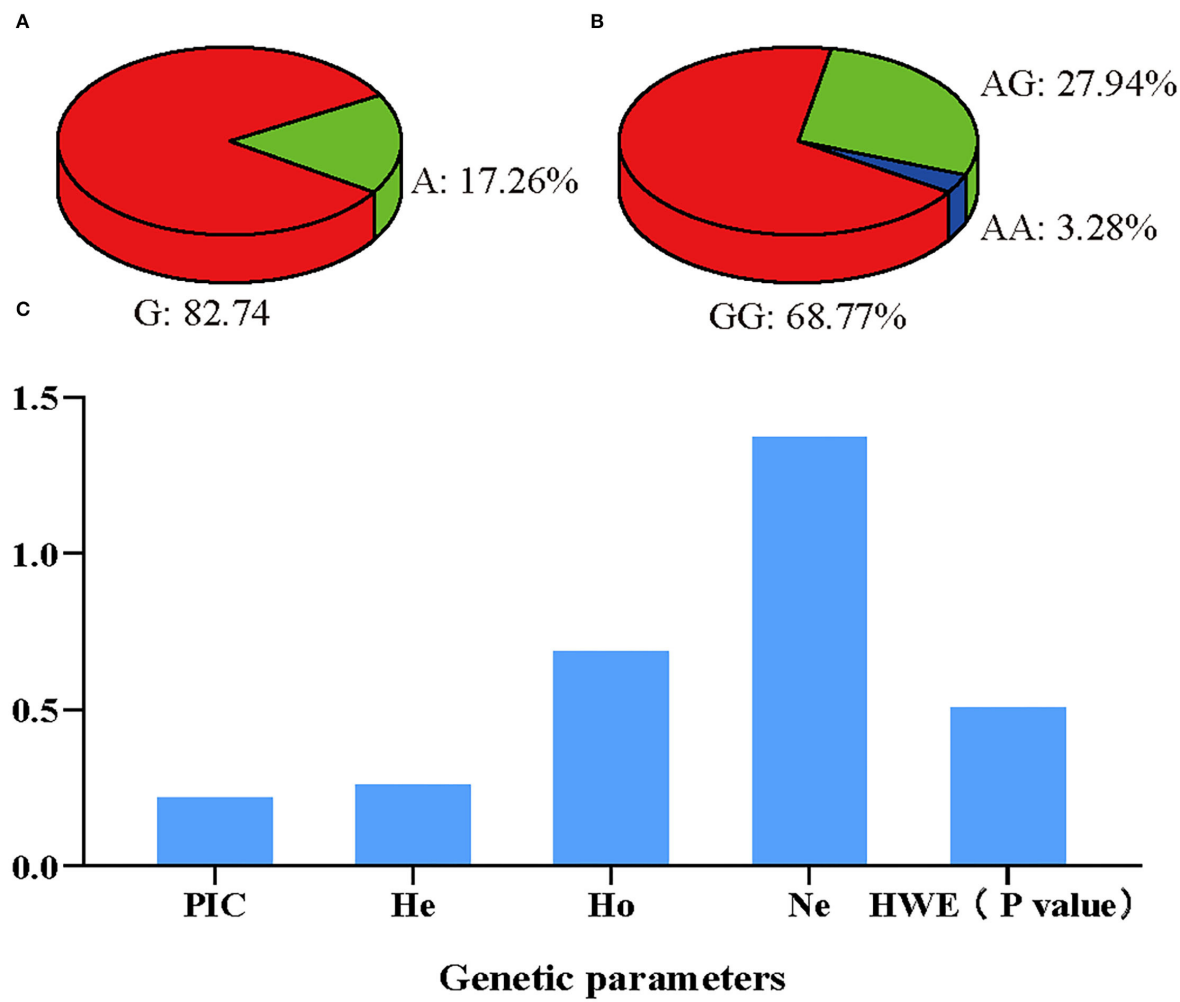
Gene frequency and genetic parameters of *IL18* g. 24991651 A > G in Hu sheep population. **(A)** Allelic frequency of *IL18* g. 24991651 A > G site. **(B)** Genotypic frequency of *IL18* g. 24991651 A > G site. **(C)** Genetic parameters of *IL18* g. 24991651 A > G site. Ne, effective allele numbers; Ho, homozygosity; He, heterozygosity; PIC, polymorphism information content; HWE, Hardy–Weinberg equilibrium.

First, in the experimental population of 96 Hu sheep, these three SNPs were genotyped by the KASP assay, and all three genotypes were generated: GG, GA, AA/GG, GA, AA/TT, TA, AA, respectively (Figures 4D–F). Then, the linkage disequilibrium analyses were performed based on the above obtained genotyping data, the result showed that the three SNPs were in a strong linkage state (Figure 5). Therefore, only the SNP was genotyped in the enlarged experimental population, and the genotypic and allelic frequencies and genetic parameters for *IL18* g. 24991651 A > G (Oar_rambouillet_v1.0) were calculated, and shown in Figure 6, the result showed that the SNP was of low polymorphisms (PIC < 0.25), and the locus was in Hardy–Weinberg equilibrium (*p* > 0.05).

### Association of Polymorphism of *IL18* Gene With Hematologic Parameters in Hu Sheep

To evaluate the effect of the polymorphism of ovine *IL18* on selected traits. The association analysis between different genotype and traits was performed by using SPSS 17.0 software. The results demonstrated that the polymorphism g. 24991651 A > G was significantly associated with RBC, MCV, MCHC, and RDW_CV, and the individuals with AA genotype had higher RBC and MCHC than the individuals with GA and GG genotypes (*p* < 0.05), while the MCV in the animals with AA genotype was lower than those of the animals carrying the GA and GG genotypes (*p* < 0.05) (Table 4).

**Table 4:** Association analysis between polymorphism of *IL18* gene and hematologic traits of Hu sheep.

	***IL18*** **g. 24991651 A** **>** **G**		
**Traits**	**GG**	**GA**	**AA**
NO.	543	221	26
RBC (M/ul)	14.21 ± 0.06^b^	14.00 ± 0.09^b^	14.76 ± 0.27^a^
HGB (g/dl)	13.16 ± 0.05	13.05 ± 0.07	13.41 ± 0.21
HCT (%)	31.19 ± 0.15	30.72 ± 0.24	30.27 ± 0.70
MCV (fl)	22.10 ± 0.12^a^	22.09 ± 0.19^a^	20.65 ± 0.57^b^
MCH (pg)	9.29 ± 0.03	9.36 ± 0.04	9.13 ± 0.13
MCHC (g/dl)	42.52 ± 0.20^b^	42.88 ± 0.32^b^	44.81 ± 0.92^a^
RDW_SD (fl)	26.55 ± 0.14	26.45 ± 0.22	27.27 ± 0.63
RDW_CV (%)	42.59 ± 0.11^ab^	42.11 ± 0.17^b^	43.42 ± 0.50^a^
WBC (K/ul)	12.21 ± 0.12	12.33 ± 0.19	11.80 ± 0.56

a, b *Values in the same row with different superscripts differ significantly (p < 0.05). For a definition of the hematologic trait abbreviations, please refer to the footnote of Table 3*.

## Discussion

In livestock breeding, health-related traits are becoming a priority, while hematologic parameters are important to evaluate the health condition, feeding management and nutrition level of animal, and the variation hematologic traits are caused by genetic and non-genetic (e.g., breed, age, sex and management systems) (24). At present, the studies on sheep hematologic parameters mainly focus on nutrition level and breed (25–27). In this study, the hematologic traits were measured for 819 male Hu sheep aged 6 months under the same nutritional level and management conditions, and the descriptive statistics and heritability estimates were performed for these traits, the results shown the average values were within the reference ranges of sheep according to the previous studies (26), the coefficient of variation were >10%, suggesting that these indexes have a certain selection space in the Hu sheep population. In addition, the five indexes presented medium-to-high heritability values; this is consistent with the study reported by Ponsuksili et al., who reported a genomic heritability estimate for hematological traits that ranged from 0.17 to 0.68 in pig (28). These results will provide the reference value for the hematological indexes in sheep.

Genetic variability of hematological indexes will directly or indirectly affect the profitability of the sheep breeding industry. Screening genetic variations of the key candidate genes related to the hematological indexes will contribute to design the breeding strategies for evaluating the animal health condition. Single nucleotide polymorphisms are served as the most effective and extensive DNA marker in the modern genetic breeding. Interleukin 18, as a immune regulator, plays a vital role in immune responses (29). In this research, *IL18* gene was used as a candidate gene to detect the expression level and identify the polymorphisms. First, we detect the expression feature of *IL8* in different tissues of Hu sheep aged 6 months, the result showed that the broad expression pattern of *IL18* gene in Hu sheep, with expression detected in all 10 of the tissues tested (heart, liver, spleen, lung, kidney, rumen, duodenum, lymph, tail fat, and longissimus dorsi), and the expression level of *IL18* was evidently higher in lymph and lung tissues compared with the others tested tissues (*p* < 0.05). Fournout et al. reported *IL18* expression levels in different organs of piglets, the results demonstrated that the expression level of *IL18* was higher in the lymph, while *IL18* is weakly expressed in the lung (30). The lymph is a main component of the animal immune system and plays a vital role in immunity system (31). Further, to evaluate the effects of the variation of *IL18* gene on hematologic indexes, three novel variation sites were found in the intronic region of *IL18* gene using DNA sequencing, and the three SNPs were in a strong linkage state. Therefore, one polymorphism site was genotyped in the enlarged experimental population. Although, the intron variation does not alter amino acid, the previous evidence suggests that the intron mutations may have similar effects to synonymous mutations, and synonymous mutations affect the gene functions by altering protein expression, conformation, mRNA stability, and structure (32, 33). Therefore, the potential association between the different genotypes and hematologic indexes were performed in this study, the result indicating the polymorphism g. 24991651 A > G was significantly associated with RBC, MCV, MCHC, and RDW_CV (*p* < 0.05), and all the phenotype measured values of RBC, MCHC, and RDW_CV in the experimental population with the AA genotype were evidently higher than those with the GA and GG genotypes (*p* < 0.05), there was no evident difference between the GA and GG genotypes. The phenotype values of MCV in the animals with the AA genotype were significantly lower than those with the population with the GA and GG genotypes (*p* < 0.05), whereas the difference between GG and GA were not significant.

The changes observed in MCHC, MCV, and RDW are probably directly associated with the reticulocytosis (34). In addition, the hematologic indexes can be used to reflect the production performance of livestock; thus, we speculate that polymorphic site in *IL18* may be regarded as candidate genetic markers for evaluating the health state and the feeding management of sheep. In spite of this, the functions of *IL18* gene need to be further explored at the cell and protein levels.

## Conclusion

In this study, the descriptive statistics and heritability of hematologic traits were estimated in male Hu lambs, revealing that the partial hematologic indexes have great potential for selection and show medium-to-high heritability. Simultaneously, the *IL18* expression features were detected in different tissues, the results indicated *IL18* expressed predominantly in lung and lymph compared with the others tested tissues. Additionally, a novel polymorphism detected in *IL18* was significantly association with RBC, MCV, MCHC, and RDW_CV. These results provide the reference information of sheep hematological parameters and identified the potential of the ovine *IL18* gene as a candidate for improving immune traits in the sheep breeding program.

## Data Availability Statement

The datasets presented in this study can be found in online repositories. The names of the repository/repositories and accession number(s) can be found in the article/Supplementary Material.

## Ethics Statement

All animal studies were performed in strict accordance with the guidelines proposed by the Regulation of the Standing Committee of Gansu People's Congress. Collection of blood and tissue samples as well as experiments procedures involved in the present study were approved by the Ethics Committee of the College of Pastoral Agriculture Science and Technology of Lanzhou University.

## Author Contributions

WW and DZ designed and conceived the experiments. DZ, XZ, FL, YuaZ, XL, JW, LZ, YukZ, JC, WL, CL, and BZ contributed to the sample and data collection. DZ, XY, LZ, and JW involved in RNA and DNA extraction. DZ wrote the manuscript. WW, XZ, and DZ reviewed and edited the manuscript. All authors have read and approved the final manuscript.

## Funding

This work was supported by the National Key R&D Program of China (2021YFD1300901), the National Joint Research on improved breeds of Livestock and Poultry (grant no. 19210365), and the Key R&D Program of Gansu Province (grant no. 20YF3NA012).

## Conflict of Interest

The authors declare that the research was conducted in the absence of any commercial or financial relationships that could be construed as a potential conflict of interest.

## Publisher's Note

All claims expressed in this article are solely those of the authors and do not necessarily represent those of their affiliated organizations, or those of the publisher, the editors and the reviewers. Any product that may be evaluated in this article, or claim that may be made by its manufacturer, is not guaranteed or endorsed by the publisher.
